# Combined Treatment with Hyaluronic Acid and Mesalamine Protects Rats from Inflammatory Bowel Disease Induced by Intracolonic Administration of Trinitrobenzenesulfonic Acid

**DOI:** 10.3390/molecules22060904

**Published:** 2017-05-30

**Authors:** Chih-Tung Chiu, Sheng-Nan Kuo, Shao-Wen Hung, Cheng-Yao Yang

**Affiliations:** 1 Holy Stone Healthcare Co., Ltd., Taipei 11493, Taiwan; davidchiu@hshc.com.tw (C.-T.C.); samuelkuo@hshc.com.tw (S.-N.K.); 2 Division of Animal Resource, Animal Technology Laboratories, Agricultural Technology Research Institute, Hsinchu City 30093, Taiwan; 1032169@mail.atri.org.tw

**Keywords:** colitis, hyaluronic acid, inflammatory bowel disease, mucosal healing, remission

## Abstract

Drugs such as mesalamine (5-ASA) are currently recommended for the treatment of inflammatory bowel disease (IBD). To reduce the frequency of their administration and improve their therapeutic effect, this study investigated the adhesion efficacy, wound healing promotion, and decrease in inflammation in ulcers in the colonic tissue of rats with colitis after combined treatment with hyaluronic acid (HA) and 5-ASA (IBD98-M). HA-fluoresceinamine (FL) conjugates successfully adhered to the mucosal layer and were conjugated in the vascular tissue. In addition, macroscopic and microscopic observations indicated that colonic injuries reduced significantly after treatment with IBD98-M. Compared with PBS and 5-ASA treatment alone, treatment with IBD98-M more effectively reduced bowel inflammation and promoted colonic mucosal healing in TNBS-induced colitis. IBD98-M treatment also reduced myeloperoxidase activity and the expression levels of cyclooxygenase 2 and tumor necrosis factor-αin the colitis tissue. In conclusion, IBD98-M treatment strongly promoted wound healing in colonic injuries and significantly inhibited MPO activity in the inflamed colon tissue of rats. Combined treatment with HA and 5-ASA can accelerate wound healing and reduce inflammatory reaction in rat colitis.

## 1. Introduction

Inflammatory bowel disease (IBD) is a multifactorial chronic disease involved in ulcerative colitis (UC) and Crohn′s disease (CD), which are chronic intestinal disorders caused by dysfunctional or abnormal epithelial and immune responses to intestinal microorganisms. The etiology of IBD remains unclear; however, both environmental and genetic factors are believed to contribute to its etiology. Because these multifactorial interactions contribute to IBD, it is a complex disease [1,2,3]. Todate, the pathogenesis of IBD has not been well elucidated. However, some cellular and molecular insights into IBD pathogenesis have been achieved. In addition, the upregulation of proteins such as cyclooxygenase-2 (COX-2), inducible nitric oxide synthase [4,5,6], and proinflammatory cytokines is involved in IBD pathogenesis and is considered a crucial determinant of colonic damage [7]. 

Mesalamine, also known as 5-aminosalicylic acid (5-ASA) is a well-established drug currently used in the first-line treatment of patients with IBD, particularly those with active mild-to-moderate UC [8]. In most clinical cases, 5-ASA was observed to rapidly and extensively absorb before reaching the colon [9]. Moreover, although 5-ASA can cause adverse side effects, it is usually well tolerated [10]. 

Hyaluronic acid (HA), also known as hyaluronate or hyaluronan, is a non-sulfated glycosaminoglycan and an endogenous high-molecular-weight linear polysaccharide with a repeating disaccharide unit (β-1,4-d-glucuronic acid and β-1,3-*N*-acetyl-d-glucosamine); HA is found in the connective tissue, synovial fluid, umbilical cord, and vitreous humor [1,2,3,11]. HA has numerous embryological and wound-healing properties [11], and it stimulates cell motility through cluster of differentiation 44 (CD44) and hyaluronan-mediated motility receptors [12,13]. Endothelial cells in inflamed lesions of Inflammatory bowel diseases have an activated phenotype and are known to express high levels of CD44 that appear to be pivotal for immune cell (such as monocytes, macrophages, neutrophils and lymphocytes) infiltrate into inflamed tissues [14,15]. Since CD44 plays a role in cell migration, modulation of CD44-HA binding by inflammatory cytokines may have profound effects on immune cell recruitment [16,17] and aid in wound repair [18]. The degradation products of HA can act as damage-associated molecular patterns to modulate the immunological response at an injury site [19]. Additionally, HA can delay or reduce the granulation of tissues and newly formed capillaries near subcutaneous implants [20]. The unique viscoelastic nature of HA along with its biocompatibility and non-immunogenicity has led to its use in numerous clinical applications [21]. Fluorescein amine (FL)-labeled HA has been prepared and used for investigating biological functions [22,23] and tissue distribution [24]. Studies have investigated the interaction of the HA biopolymer with its binding proteins (e.g., CD44) by using HA-FL biomaterials [22,23].

Intracolonic administration of 2,4,6-trinitrobenzenesulfonic acid (TNBS) is one of the standardized methods used to develop an experimental model of IBD, which closely mimics the clinical and morphological features of IBD, particularly CD [8]. In this model, ethanol is used to remove the protective mucus coat, and TNBS is used as an enema to induce chronic colitis [1,2,3]. Despite improvements in treatment modalities, the current status of drug therapy and therapeutic strategies for IBD is not satisfactory. In this study, we assessed colon inflammation by determining colon weight, length, weight/length ratio, macroscopic lesions, microscopic score, and leukocyte invasion—as indicated by myeloperoxidase (MPO) activity. In addition, we investigated inflammatory effects by examining the mRNA expression levels of TNF-α, IL-1β, and IL-6 [25,26]. The search for novel IBD therapeutic compounds and the development of IBD therapeutic strategies are ongoing challenges. An alternative medicine with high efficacy and fewer adverse effects is urgently required. Thus, in this study, we attempted to gain a better understanding of the therapeutic effects and mechanisms of actions during early colonic inflammation in a TNBS-induced rat model of IBD.

## 2. Results

### 2.1. Ex Vivo Adhesive Properties of FL-Labeled HA Conjugates in the Colon Tissue of Rats

**Figure 1:**
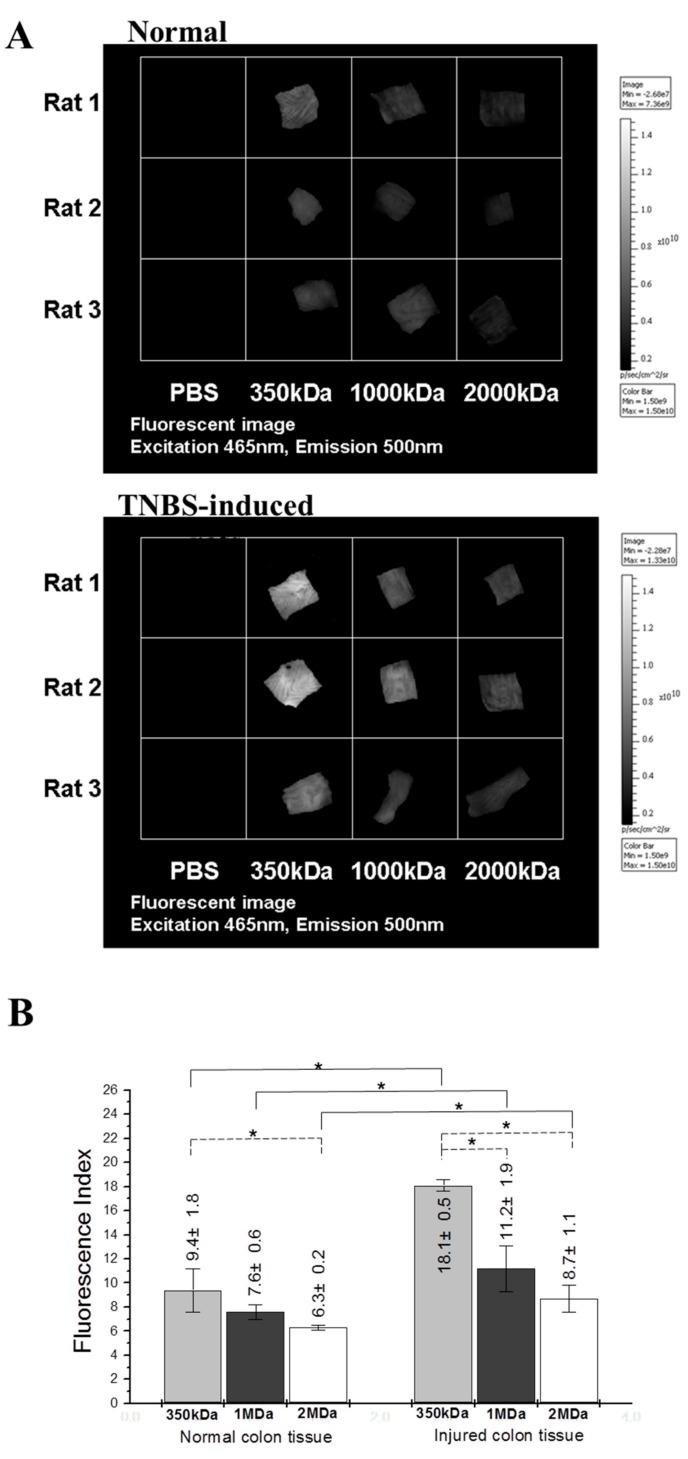
Adhesion observations of three FL-Labeled HA conjugates in the colon tissues of rats by using an IVIS. Three FL-Labeled HA conjugates used in the normal and injured colon tissues of rats (**A**); Three FL-Labeled HA conjugates used in the normal (upper) and injured (below) colon tissues of rats. Quantitative analysis of fluorescence intensity (%) = intensity of the tissue immersed in the sample solution/intensity of the tissue immersed in PBS solution (**B**). Data represent the mean ± SEM (*n* = 6) of three independent experiments. Differences were considered significant if the *p* < 0.05 (*).

The colon lumen tissue was physically damaged using a toothbrush, and a non-invasive in vivo imaging system (IVIS) was used to observe the FL-Labeled HA conjugates adsorbed on the mucosal layer of the colon. The normal and injured colon tissues immersed in phosphate buffer saline (PBS) solution exhibited the same non-fluorescent imaging and low intensity (Figure 1A). The adhesion characterization assay is based on the following formula: fluorescence index (%) = intensity of the tissue immersed in a sample solution/intensity of the tissue immersed in PBS solution. To quantify fluorescence intensity, data were organized (Figure 1B) to obtained the fluorescence index. The quantitative results revealed that the fluorescence intensity of the injured colon tissue immersed in FL-Labeled HA conjugates was higher than that of the normal colon tissue. This finding indicates that the adhesion of FL-Labeled HA conjugates was higher on the injured tissues than on the normal tissues, particularly with the HA (350 kDa)-FL conjugate (Figure 1B). In addition, regarding the molecular weight (350 kDa, 1000 Da, and 2000 Da), the HA (350 kDa)-FL conjugate appeared to have better adhesion properties compared with HA (1000 kDa)-FL and HA (2000 kDa)-FL conjugates in the normal and injured colon tissues of rats.

These results demonstrate that the amount of the HA biopolymer adsorbed on the injured colon tissue was significantly higher than that on the normal colon tissue (*p* < 0.05 (*); Figure 1B). The 350 kDa HA and 2000 kDa were choose in IBD98-M formulation for adhesion and viscosity properties respectively [27].

### 2.2. In Vivo Adhesive Observation of FL-Labeled HA Conjugates on the Colon Epithelia

**Figure 2:**
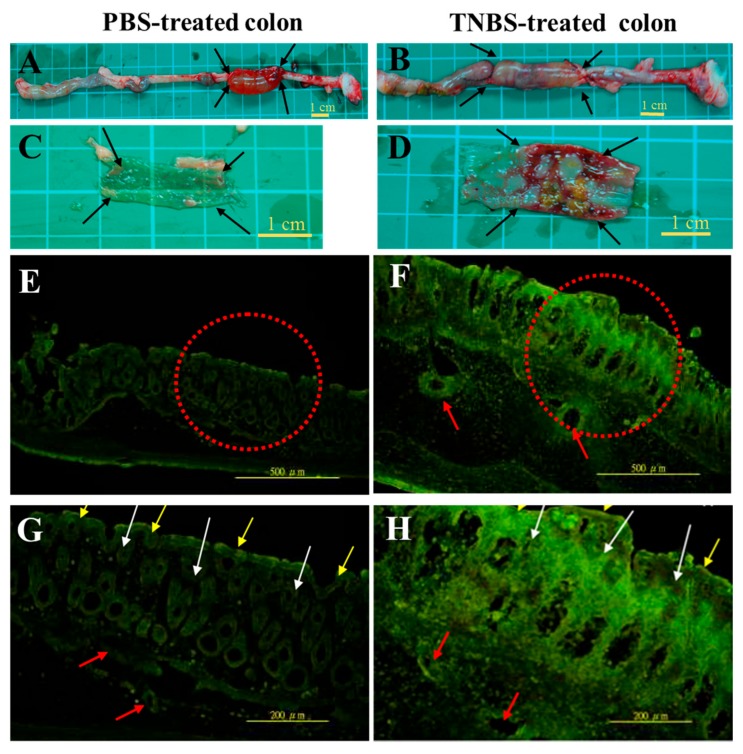
The adhesion of FL-Labeled HA conjugateson the normal and ulcerous mucosa of rat′s-colons. The HA-FL conjugate is more adhesive on the ulcerous mucosa than on the normal colon. The mucosal tissue of normal rats is shown in panels **A**, **C**, **E** and **G**. The ulcerous tissue of rats with colitis is shown in panels **B**, **D**, **F** and **H**. Black arrows indicate the macroscopic appearance of a normal colon (**A**) and an ulcerous colon (**B**). The inner appearances of a normal colon (**A**) and an ulcerous colon (**B**) are presented in panels **C** and **D**, respectively. The fluorescence expression of a colon tissue cryosection [Figure **E** and **F** is at 10× magnification (red dashed cycle); Figure **G** and **H** is at 200× magnification]. Yellow arrows, white arrows, and red arrows indicate the mucosal layer, chorionic villi, and vascular vessels, respectively.

**Figure 3:**
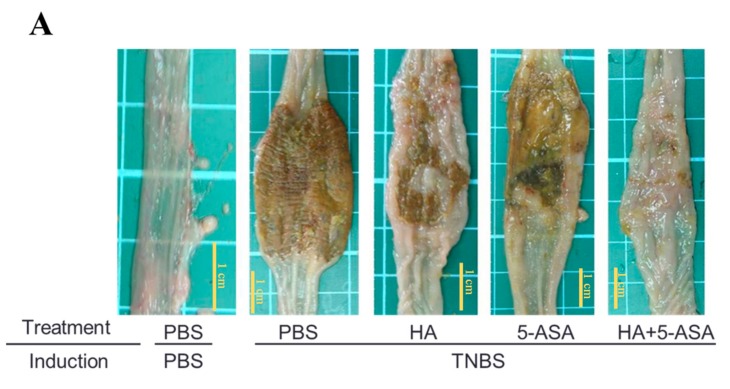

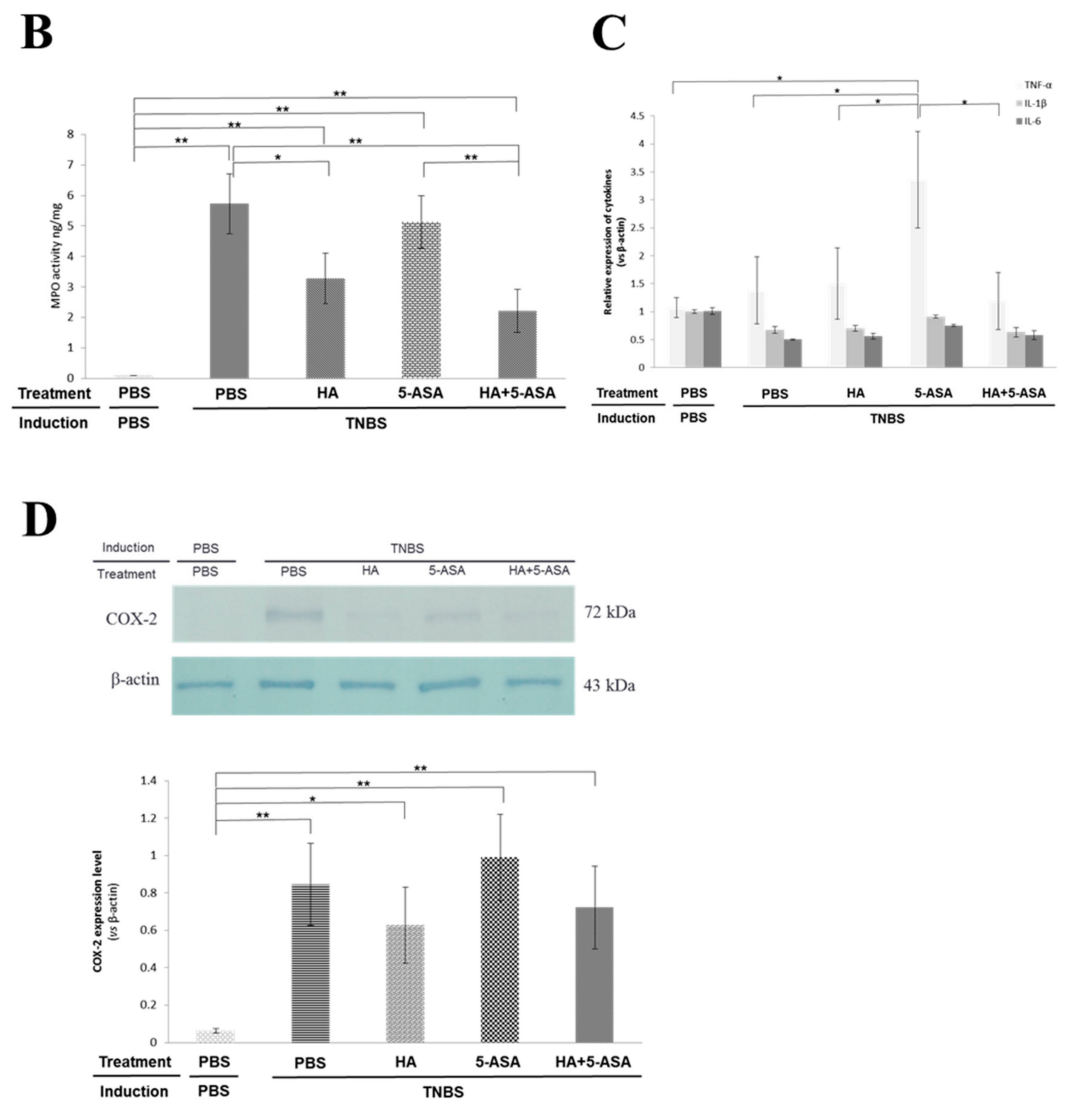
The macroscopic inner appearance, MPO activity, and proinflammatory cytokine and COX-2 expressions in the colon tissues of rats following induction (PBS or TNBS) and treatment (PBS, HA, 5-ASA, and HA + 5-ASA, respectively). According to the macroscopic inner appearance of the colon, combined treatment with HA and 5-ASA exerts a synergistic protective effect on TNBS-induced colitis compared with other treatments following TNBS induction (**A**). Combined treatment with HA and 5-ASA exerts the synergistic effects of suppression of MPO activity on TNBS-induced colitis compared with other treatments following TNBS induction (**B**) Related expression of mRNA of TNF-α, IL-1β and IL-6 genes represent the mean ± SE. Unlike IL-1β and IL-6, TNF-α gene expression more significantly decreased after combined treatment with HA and 5-ASA than after 5-ASA treatment alone in TNBS-induced colitis (**C**); Data represent the mean ± SE. COX-2 expression significantly decreased after HA treatment alone and combined treatment with HA and 5-ASA compared with either treatment alone (PBS or 5-ASA) in TNBS-induced colitis (**D**); Each bar represented the mean ± SE. The bars with different star were significant different (*p* < 0.05 (*), *p* < 0.01 (**)) from each other.

Compared with that of healthy rats, the colon tissue of rats with colitis exhibited a significant decrease in total length and a significant increase in thickness. The arrows in Figure 2A,B indicate the location where in situ excisions were performed; these can also be observed in Figure 3A. The colon tissues (Figure 2A,C) and potential ulcers (Figure 2B,D) of healthy rats and rats with colitis were visualized on a translucent film. Higher-magnification images are shown in Figure 2E,F (100× magnification) and Figure 2G,H (200× magnification).

In Figure 2E–H, the mucosal epithelial layer (yellow arrow), chorionic villi (white arrow), and vascular tissue (red arrow) were identified by green fluorescence in the cryosection of colon tissues. In the healthy colon tissues, the boundary layers between mucosa, chorionic villi, and vascular tissue were clear. The lamina propria layer exhibited minor macrophage infiltration. FL-Labeled HA conjugates adhered to the mucosal layer and were conjugated in the vascular tissue. The mucosal layer and chorionic villi were both damaged, and the submucosal layer was thicker in the groups that underwent TNBS induction than in the normal control group. Furthermore, many macrophages assembled in the lamina propria and submucosal layer of the colon of rats.

### 2.3. Clinical Findings and Macroscopic and Cumulative Microscopic Assessment of Rats with TNBS-Induced Colitis

Sprague Dawley (SD) rats were intracolonically administered TNBS to induce colitis, and intracolonic treatments were performed with PBS, HA, 5-ASA, and a combination of HA and mesalamine (5-ASA). PBS-induced rats with PBS treatment served as the normal control. Data revealed that SD rats developed colitis after TNBS induction. Additionally, soft stools or diarrhea and slow weight gain were observed in rats with colitis. After individual treatment on day 3, PBS-induced rats with PBS treatment exhibited significant weight gain compared with rats in other treatment groups. By contrast, rats with colitis in other treatment groups (PBS, HA, 5-ASA, and combined HA and 5-ASA) exhibited only slight weight gain after 3 days of treatment. The weight gain of HA-treated rats (HA alone treatment or combined treatment with HA and 5-ASA) was higher than that of rats not treated with HA (PBS or 5-ASA treatment; Table 1). The trends of stool consistency and lesions were similar to that of weight gain among the groups. The bowel wall thickness of rats that underwent combined treatment with HA and 5-ASA was significantly lower than that of rats that underwent treatment with PBS, HA, or 5-ASA (Table 1).

Macroscopic injuries in the colon of rats were observed on day 3 after TNBS induction and individual treatments. After sacrificing the SD rats, the region between the colorectal junction and the anus was removed, and a longitudinal incision was made. Data revealed that the length of the inflamed colon decreased by 3 cm compared with the normal colon. Colitis severity was characterized by bowel wall thickness, inflammatory area, and macroscopic lesions. The gross lesions of the colon were more severe in rats that underwent PBS and 5-ASA treatments than in rats that underwent HA treatment alone and combined treatment with HA and 5-ASA; these results are in agreement with the trend in clinical findings (Figure 3A, Table 1). In addition, these data supported that the combined treatment with HA and 5-ASA exerted a better effect on UC than did HA or 5-ASA treatment alone (Figure 3A, Table 1). 

Microscopic injuries in the colon of rats were observed on day 3 after TNBS induction and individual treatments. Histopathological assessment of colitis was performed. The cumulative microscopic colitis injury score for rats on day 3 after TNBS induction was 8.33 ± 0.49, which represented moderately severe colitis. Moreover, the colitis injury scores after 5-ASA treatment alone and after combined treatment with HA and 5-ASA were 6.33 ± 0.61 and 7.27 ± 0.21, respectively; the treatments did not significantly reduce the injury scores in these groups compared with the control group that underwent TNBS induction and PBS treatment. However, the HA-treated group exhibited a significant reduction in colitis injury (*p* < 0.001) compared with the control group that underwent TNBS induction and PBS treatment. These data indicate that HA treatment significantly reduced the degree of inflammatory cell infiltration and colon ulceration (Table 1).

### 2.4. Suppression of MPO Activity and TNF-α Gene and COX-2 Protein Expressions after the Combined Treatment with HA and 5-ASA in Rats with TNBS-Induced Colitis

Evaluation of the MPO activity is crucial to understand the effects of the HA biopolymer and/or 5-ASA on SD rats with colitis. The MPO activity in the colonic tissues of SD rats that underwent PBS, HA, or 5-ASA treatments alone on day 3 after TNBS induction was 5.72 ± 0.98, 3.28 ± 0.82, and 5.13 ± 0.85 ng/mg, respectively. The combined treatment with HA and 5-ASA significantly reversed the effect of the 5-ASA treatment alone (*p* < 0.05), and the MPO activity decreased from 5.13 ± 0.85 ng/mg to 2.22 ± 0.71 ng/mg in the tissues (Figure 3B).

The pro-inflammatory cytokine mRNA expression in the colon of SD rats was determined through real-time polymerase chain reaction (PCR). The results revealed no significant difference in TNF-α gene expression under HA treatment alone compared with control PBS treatment combined treatment (HA + 5-ASA) and PBS/PBS treatment. The combined treatment with HA and 5-ASA significantly reversed the effect of treatment with 5-ASA alone (*p* < 0.05). In addition, the mRNA levels of IL-1β and IL-6 did not significantly differ among any of the groups (Figure 3C). Therefore, the mRNA levels of IL-1β and IL-6 are not suitable as post-treatment indicators in this study.

The expression level of COX-2 was examined using western blot analysis. The data revealed high protein expression of COX-2 in the colon tissue of SD rats on day 3 after TNBS treatment (Figure 3D). Treatment with HA alone significantly reduced COX-2 expression; however, 5-ASA treatment alone did not significantly reduce COX-2 expression. Additionally, combined treatment with HA and 5-ASA reduced COX-2 protein levels to one-third compared with that observed after treatment with 5-ASA alone (Figure 3D).

## 3. Discussion

The normal mucus layer, approximately 800 μm in thickness, provides a protective barrier between the underlying epithelium and lumen, which contain noxious agents, destructive hydrolases, and microorganisms [1,2,3]. The functional efficacy of the adherent mucus layer depends on its thickness and stability in vivo as well as the physical and chemical properties of the gel. In inflamed colon tissue, the mucus layer is not sufficient to protect against microorganisms in the area of an ulcer [1,2,3]. In this study, HA biopolymers were evaluated for their adhesive properties with respect to the mucus layer. FL-Labeled HA conjugates exhibited stronger adhesion in the ulcerous tissue than in the healthy colon tissue. Thus, these materials supplement the viscous mucus that prevents the mucosa from a repeat injury.

The HA content of blood increased with time following the induction of dextran sulfate sodium (DSS) in mice. Ulcer tissues may need more HA biopolymers during the wound healing process [10,28]. Moreover, studies have demonstrated that mice with colitis responded more favorably to treatment with HA biopolymers than to sham treatment [10]. In clinical research, HA biopolymers have been used as a strong adhesive material for oral ulcers associated with Behcet’s disease; the suggested dose for treatment is 0.2% of HA gel [29,30]. Therefore, HA biopolymers are functional in the wound healing of ulcers. In this study, we successfully demonstrated the application of a rat model with colitis to evaluate the treatment effects of HA biopolymers, 5-ASA, and IBD98-M. The results indicated that the treatment of TNBS-induced colitis with IBD98-M through daily rectal administration significantly reduced colonic inflammation, mononuclear infiltration, and ulceration and accelerated mucosal healing. With respect to findings regarding the gross lesions, bowel wall thickness, inflamed areas in the colon mucosa, and macroscopic scores of colitis, the results obtained after IBD98-M treatment were more satisfactory than those obtained after other treatments alone. 

TNBS-induced colitis mimics human IBD with respect to several histological alterations, including the mucosal invasion of polymorphonuclear cells (as indicated by MPO activity) and contributions to colon injury [4,31]. Treatment with IBD98-M significantly inhibited MPO activity in TNBS-treated rats. HA attenuated leucocyte influx to the inflamed colon, as shown by macroscopic lesions, histopathology, and diminished MPO activity. These observations are in accordance with those of previous studies [4,32,33]. However, treatment with 5-ASA alone did not inhibit MPO activity or cytokine gene expression, which may have been due to insufficient residence time in the colon cavity because 5-ASA was dissolved in PBS solution. HA biopolymers protect mucosal cells and anatomical structures against chemical wounds through their viscoelastic characteristics [34]. Treatment with IBD98-M exhibited similar trends; the combination substantially healed injuries in the mucosal area and resulted in macroscopic changes and changes in COX-2 and TNF-α expressions. However, treatment with 5-ASA alone did not result in the same trends. These data indicate that intraluminal therapy with IBD98-M is better than treatment with either PBS or 5-ASA alone. Satisfactory effects involving reduced bowel inflammation and promotion of mucosal repair were found in rats treated with IBD98-M.

Numerous pro-inflammatory cytokines are involved in regulating wound repair, including TNF-α, IL-1β, and IL-6 [35]. TNF-α produced by macrophages and endothelial cells is necessary for the initiation and persistence of TNBS-induced colitis [35]. TNF-α-mediated inflammation in vascular smooth muscle cells is believed to be a major mechanism underlying the pathophysiology of atherosclerosis [36,37]. IL-1β and IL-6 are crucial in IBD and arrest DSS-induced colitis by inhibiting the pro-inflammatory activity and possible downstream proangiogenic activity. All the aforementioned factors have been identified in IBD and experimental colitis [10]. Although the gene expression levels of IL-1β and IL-6 did not show a significant difference between the groups, TNF-α gene expression was more significantly suppressed after IBD98-M treatment than after 5-ASA treatment alone, which might contribute to the suppression of the inflammatory response in colitis.

COX-2 is a key enzyme in the conversion of arachidonic acid to prostaglandins. Recently, a study suggested that COX-2 plays a critical role in maintaining mucosal homeostasis [38]. In addition, COX-2 is upregulated in colitis, generating various products implicated in oxidative damage and inflammation [29]. In this study, our data demonstrated that treatment with IBD98-M reduced the COX-2 expression level, which might contribute to the suppression of the inflammatory response in colitis. Although our results were revealed that the combined treatment with HA and 5-ASA can accelerate wound healing and reduce inflammatory reaction in rat colitis compared with PBS alone and 5-ASA alone treatment. However, except cumulative microscopic injury represent significant difference between the combined treatment and HA alone treatment, our other results were not revealed significant difference between the combinatory treatment and HA alone treatment. Therefore, the dosage forms and treatment strategies of HA and 5-ASA conjugates should be required further assessment and design. We hope that the modified dosage forms of HA and 5-ASA conjugates may have more great therapeutic potential for the patient with IBD in the future.

## 4. Materials and Methods

### 4.1. Experimental Reagents

HA in sodium salt form were purchased from Shandong Freda Biochem Co., Ltd. (Jinan City, Shandong, China). Mesalamine was purchased from Chemi SPA (Patricia, Italy). Zoletil 50 was purchased from Virbac (Taiwan) Co., Ltd. (Taipei city, Taiwan). All other experimental reagents were obtained from Sigma-Aldrich (St. Louis, MO, USA). IBD98-M, which is a mixture of 5-ASA and HA, was prepared in our laboratory. 

### 4.2. Preparation of Fluorescent HA Conjugates

First, 125 mg of HA powder was completely dissolved in PBS solution. Solution A was prepared by dissolving 65 mg of FL powder in 95% EtOH solution. Solution B was prepared by dissolving 359 mg of 1-ethyl-3-(3-dimethylaminopropyl) carbodiimide in MES buffer. Solution C was prepared by dissolving 216 mg of N-hydroxysuccinimide in MES buffer. Then, 3 mL of solution A was added into 0.5% HA solution. Subsequently, 3 mL of solution B and 5 mL of solution C were separately added into the reaction solution. Finally, 0.02 M MES buffer was added to the reaction solution, which was then dialyzed with distilled deionized water by using a dialysis membrane (Apectra/ProR Dialysis Membrane; molecular weight: 12,000–14,000; Spectrum Laboratories, Rancho Dominguez, CA, USA) at 4 °C for 5 days until it exhibited no fluorescence. The final product was dehydrated using a freeze-drying process, and the FL-Labeled HA conjugates were stored at −20 °C.

### 4.3. Animal Care

All animal experiments were approved by the Institutional Animal Care and Use Committee of the Animal Technology Laboratories, Agricultural Technology Research Institute, Miaoli, Taiwan, and animal care was performed in compliance with guidelines of the European Union regarding animal experimentation. Eight-week-old male SD rats (200–250 g) were obtained from BioLASCO Taiwan Co., Ltd., Taipei city, Taiwan. The rats were housed two per cage under a 12-h light/dark cycle at 23 °C–25 °C and 70–75% humidity. Normal laboratory diet (Panlab, Barcelona, Spain) and fresh water were supplied to rats continuously ad libitum.

### 4.4. Ex Vivo Adhesion of FL-Labeled HA Conjugates in Colon Tissue through Observation with an IVIS

Normal colon tissues were obtained from SD rats (*n* = 3/group). Physical injury was induced in the colon tissues by lightly brushing them lengthwise approximately 20 times. The colon tissues were immersed in 0.25% HA-FL solution for 1 h at room temperature. Observations were then conducted using a non-invasive in vivo imaging system (IVIS^®^ Imaging System 200 Series, Xenogen Co., Alameda, CA, USA) at excitation and emission wavelengths of 465 and 500 nm, respectively. Images were captured using Living image software.

### 4.5. In Vivo Adhesion Observation of HA-FL in the Colon Tissue

We injected 0.25% FL-Labeled HA conjugates solutions into the injured area for 1 h after TNBS treatment for 24 h. After 24 h, the body weight and stool consistency of the rats treated with TNBS were examined. Then, 12 colitis rats and 12 normal rats were deeply anesthetized, and their rectum and distal colon were identified through an abdominal midline incision. To retain the FL-Labeled HA conjugates in the distal colon cavity, the distal colon was knotted at 6 cm and 8 cm from anus. Furthermore, 1 mL of the FL-Labeled HA conjugates was injected into the ligated area of the distal colon. One hour after the injection, the animals were sacrificed using an overdose of an anesthetic agent. The rectum and distal colon were excised to determine the total colon length and thickness. The ligated area of the colon tissue was cut using a surgical scissor. The tissues were washed with PBS six times (10 min each time) and then immured in 4% PFA overnight. A 14-μm-thick tissue cryosection was obtained using a cryostat microtome (CM3050S, Leica, Wetzlar, Germany). The tissue section was observed through fluorescence microscopy (Olympus BX51, Tokyo, Japan; excitation: 488 nm; emission: 520 nm; magnification to 100× and 200×).

### 4.6. Induction of Colitis and Determination of Clinical Scores of Stool Consistency

The induction protocol for colitis used in this study involved a slight modification of a previously published protocol [39]. Seventy-four rats were randomly divided into 5 groups (Table 1). Briefly, SD rats were deprived of food for 24 h prior to colitis induction. A single dose of TNBS, 30 mg of which was dissolved in 0.6 mL of 50% ethanol (*w*/*v*), was injected into the anus to induce colitis. After 24 h, rats dosed initially with TNBS were randomly assigned to one of the four treatment groups to receive daily intracolonic therapy in a manner similar to that used for TNBS administration. Rats in the placebo group were treated with PBS daily. The TNBS-treated rats received one of the following treatments: PBS alone, 0.0625% HA alone, 35 mg/kg 5-ASA alone, or 0.0625% HA combined with 35 mg/kg 5-ASA. The rats were treated for 3 days and then sacrificed. During the study, the body weight and stool consistency of the rats were recorded daily. The scores of stool consistency were defined as follows: score 1, normal stool; score 2, loose stool; and score 3, watery diarrhea.

### 4.7. Assessment of the Severity of Colitis

Lesions and gross damage in the colonic mucosa were assessed. The length, weight, and thickness of the wound area on the excised colon were measured and photographed. The area of colonic ulcers was measured using Image J software. The individual features of damage caused by colitis were graded according to the method described [39]. Colonic tissue samples were taken from macroscopically damaged areas, processed for histology, and frozen in liquid nitrogen for subsequent cytokine and MPO activity measurements.

### 4.8. Histological Assessment of Colitis

The colon injury scores were defined according to the method described [39] as follows: score 0, normal; score 1, one area of inflammation and no ulcer; score 2, one ulcer; score 3, one area of inflammation and one or two ulcers; score 4, one area of inflammation and ulcers >2; score 5, two areas of inflammation and ulcers >2; and score 6, ulceration >2 cm. 

### 4.9. Assessment of MPO Activity

MPO activity was assessed as a marker of neutrophil infiltration by using the technique described by Siddiqui et al but with a slight modification [39]. Briefly, colonic tissue samples stored at −80 °C were removed and thawed on ice. After the thawing of samples, 1 mL of 0.5% hexadecyltrimethylammonium bromide (Sigma-Aldrich) containing 50 mM KH_2_PO_4_ (Sigma-Aldrich) and 0.1 M Na_2_HPO_4_ (Sigma-Aldrich) was added per 100 mg of the tissue sample for homogenization. Homogenates were centrifuged at 12,000 *g* for 10 min at 4 °C after four freeze/thaw cycles. The supernatant was collected for the MPO activity assay. A stock solution of horseradish peroxidase (0.5 mg/mL; Sigma-Aldrich) was used as a standard. Tetramethylbenzidine (TMB; Sigma-Aldrich) was used as a substrate in the MPO activity assay to initiate the reaction. In the assay, 10 μL of the standard and sample were added to appropriately labeled tubes. TMB was added at a volume of 100 μL to initiate the reaction, and 100 μL of 0.1 M H_2_SO_4_ was added after 10 min of initiation to terminate the MPO reaction. Changes in absorbance were measured using a spectrophotometer at 450 nm; MPO activity is expressed as nanogram per milligram of tissue. 

### 4.10. Analysis of Gene Levels of Proinflammatory Cytokine Expressions in Colon Tissues through Real-Time PCR

Total RNA extraction, cDNA synthesis, and gene expression analyses were performed. RNA extraction (RNA extraction mini kit, Qiagen, Hilden, Germany) from the colonic tissue was performed according to the manufacturer′s protocol. The primers and FAM-labeled probe sequences of TNF-α, IL-1β, IL-6, and β-actin used in this study are listed in Table 2. 

Real-time PCR amplification was performed using the Maxima Probe qPCR master mix (Thermo Fisher Scientific Inc., Waltham, MA, USA) with an ABI Prism 7500 fast instrument (Applied Biosystems, Foster city, CA, USA). Briefly, primers of target genes, probes, and cDNA samples were added into the Maxima Probe qPCR master mix. The reactions were performed by pretreatment with uracil-DNA glycosylase at 50 °C for 2 min and an initial preincubation at 95 °C for 10 min, followed by 40 amplification cycles, as follows: 95 °C for 15 s, 60 °C for 30 s, and 72 °C for 30 s. The gene expression levels of the genes of interest were normalized to β-actin, and the results were analyzed according to the ΔΔCt method. We reported the copy number change of the therapy group compared with that of the control group.

### 4.11. Analysis of COX-2 Protein Expression

Colonic tissue samples (250 mg) were homogenized, and the supernatant was collected as previously described. Protein concentrations were determined using the Bradford assay. Aliquots of supernatants containing equal amounts of protein (50 μg) were separated through sodium dodecyl sulfate-polyacrylamide gel electrophoresis and transferred onto a polyvinylidene difluoride membrane. The membrane was then incubated for 2 h with a polyclonal COX-2 or β-actin antibody (1:1000). The bound primary antibody was detected using an alkaline phosphatase-conjugated antirabbit antibody (1:5000), and membranes were developed using the BCIP-NBT detection system. Blot results were analyzed using the GeneTools gel analysis software. Density is expressed as gray values in densitometry units.

### 4.12. Statistical Analysis

Statistical analysis was performed using one-way ANOVA and the LSD multiple comparison test as a post hoc test. Group comparisons were performed using the nonparametric rank-based Mann–Whitney U test and the chi-square test. Differences between groups were considered statistically significant at *p* < 0.05 (*) and *p* < 0.01 (**).

## 5. Conclusions

FL-Labeled HA conjugates were successfully prepared in this study. Compared with PBS and 5-ASA treatment alone, treatment with IBD98-M more effectively reduced bowel inflammation and promoted colonic mucosal healing in TNBS-induced colitis. Moreover, IBD98-M treatment exerted a synergistic wound-healing effect on the colonic mucosa compared with HA or 5-ASA treatment alone. All treatments with IBD98-M exhibited similar trends regarding the reduction of MPO activity and COX-2 expression levels. IBD98-M may reduce injuries during colitis through inhibiting COX-2 and TNF-α expression. IBD98-M could be highly efficacious in promoting wound healing during the treatment of colitis.

## Figures and Tables

**Table 1:** Clinical findings and macroscopic and cumulative microscopic assessment of rats with colitis following induction (PBS or TNBS) and treatment (PBS, HA, 5-ASA, and HA + 5-ASA).

Group		Clinical Findings		Macroscopic Lesions					Cumulative Microscopic Injury Scores
(Induction/Treatment)	No. of Animals	Weight Gain (%)	Stool Lesion Score	Length of Colon (cm)	Weight of Colon (g)	Bowel Wall Thickness (μm)	Inflammatory Area (cm^2^)	Macroscopic Scores	
PBS/PBS	*n* = 10	14.5 ± 1.4 ^b^	1.0 ± 0 ^a^	20.4 ± 1.6 ^a^	2.2 ± 0.2 ^a^	177.2 ± 19.1 ^a^	0 ± 0 ^a^	0 ± 0 ^a^	0.0 ± 0.0 ^a^
TNBS/PBS	*n* = 16	2.4 ± 2.2 ^a^	2.6 ± 0.1 ^b^	16.6 ± 1.6 ^b^	3.2 ± 1.0 ^b^	599.1 ± 181.3 ^c^	3.39 ± 0.73 ^b,c^	1.94 ± 0.39 ^c^	8.33 ± 0.49 ^c^
TNBS/HA	*n* = 16	4.6 ± 1.5 ^a^	2.0 ± 0.2 ^c^	16.9 ± 1.7 ^b^	3.0 ± 1.0 ^b^	514.6 ± 133.7 ^b,c^	3.02 ± 0.77 ^b,c^	1.69 ± 0.36 ^b^	5.80 ± 0.58 ^b^
TNBS/5-ASA	*n* = 16	3.7 ± 1.7 ^a^	2.5 ± 0.2 ^b,c^	17.2 ± 1.4 ^b^	3.5 ± 1.1 ^b^	581.1 ± 214.7 ^c^	4.30 ± 0.70 ^c^	2.19 ± 0.33 ^b,c^	6.33 ± 0.61 ^b,c^
TNBS/HA + 5-ASA	*n* = 16	5.4 ± 1.5 ^a^	2.0 ± 0.2 ^c^	16.8 ± 1.2 ^b^	3.1 ± 0.9 ^b^	442.9 ± 98.6 ^b^	2.50 ± 0.63 ^b^	0.94 ± 0.30 ^b^	7.27 ± 0.21 ^c^

Doses of 0.0625% HA and 35 mg/kg of 5-ASA were used in this study. All values are the mean ± SE. Groups with induction (PBS or TNBS) and treatment (PBS, HA, 5-ASA, or HA + 5-ASA) in the clinical findings and macroscopic and cumulative microscopic assessment are according to the method described [39] and statistically analyzed. Differences in the clinical findings and macroscopic and cumulative microscopic assessment are indicated by the letters a, b, and c. Groups with the same letter are not statistically different.

**Table 2:** Primers and probes of proinflammatory cytokines (TNF-α, IL-1β, and IL-6) for real-time PCR.

Cytokines		Sequence	Amplicon Length	Reference
TNF-α	Primer	5′-AAATGGGCTCCCTCTCATCAG TTC-3′	110 bp	[25]
		5′-TCTGCTTGGTGGTTTGCTACG AC-3′		
	probe	FAM-CCA GAC CCT CAC ACT CAG-MGB		
IL-1β	Primer	5′-CACCTCTCAAGCAGAGCA CAG-3′	78 bp	[25]
		5′-GGGTTCCATGGTGAAGTC AAC-3′		
	probe	FAM-AGG AAA CAG CAA TGG-MGB		
IL-6	Primer	5′-TCCTACCCCAACTTCCAATGC TC-3′	78 bp	[25]
		5′-TTGGATGGTCTTGGTCCTTAG CC-3′		
	probe	FAM-AGTT TAG AGT CAC AGA AG-MGB		
β-actin	Primer	5′-CCTTCCTGTGCATGGAGT CCT-3′	202 bp	[40]
		5′-GGAGCAATGATCTTGATC TTC-3′		
	probe	FAM-AGA CCT GTA TGC CAA C-MGB		
